# Altered Negative Unconscious Processing in Major Depressive Disorder: An Exploratory Neuropsychological Study

**DOI:** 10.1371/journal.pone.0021881

**Published:** 2011-07-06

**Authors:** Zhi Yang, Jinping Zhao, Yi Jiang, Chunbo Li, Jijun Wang, Xuchu Weng, Georg Northoff

**Affiliations:** 1 Key Laboratory of Behavioral Science, Institute of Psychology, Chinese Academy of Sciences, Beijing, China; 2 School of Life Science and Technology, University of Electronic Science and Technology of China, Chengdu, China; 3 Key Laboratory of Mental Health, Institute of Psychology, Chinese Academy of Sciences, Beijing, China; 4 Shanghai Mental Health Center, Shanghai Jiao Tong University School of Medicine, Shanghai, China; 5 Center for Human Brain Research, Hangzhou Normal University, Hangzhou, China; 6 Institute of Mental Health Research, University of Ottawa, Ottawa, Canada; Royal Holloway, University of London, United Kingdom

## Abstract

**Objective:**

Major depressive disorder (MDD) has been characterized by abnormalities in emotional processing. However, what remains unclear is whether MDD also shows deficits in the unconscious processing of either positive or negative emotions. We conducted a psychological study in healthy and MDD subjects to investigate unconscious emotion processing and its valence-specific alterations in MDD patients.

**Methods:**

We combined a well established paradigm for unconscious visual processing, the continuous flash suppression, with positive and negative emotional valences to detect the attentional preference evoked by the invisible emotional facial expressions.

**Results:**

Healthy subjects showed an attentional bias for negative emotions in the unconscious condition while this valence bias remained absent in MDD patients. In contrast, this attentional bias diminished in the conscious condition for both healthy subjects and MDD.

**Conclusion:**

Our findings demonstrate for the first time valence-specific deficits specifically in the unconscious processing of emotions in MDD; this may have major implications for subsequent neurobiological investigations as well as for clinical diagnosis and therapy.

## Introduction

Emotion processing operates on a conscious level as well as in an unconscious (e.g., implicit and automatic) mode, with both being associated with different neurobiological pathways [1]–[3]. A large body of literature has focused on the conscious aspect of emotion processing as for instance in studies on emotional-cognitive regulation and its abnormalities (e.g., [4]–[5]). In contrast, the unconscious aspect has been considered as the perception and earlier processing of the emotion that precedes their cognitive regulation [6]. What remains unclear though is whether especially these different steps in emotion processing, i.e., early (unconscious) and late (conscious), are valence-specific and thus different for positive and negative emotions in healthy subjects (for reviews, see [7]).

The situation is even more complicated in patients with major depressive disorder (MDD) who suffer from an abnormal imbalance between positive and negative emotions (see [8] for a recent review). MDD can be characterized by deficits in conscious emotion processing as it is, for instance, required in regulation of especially negative emotions (see [4] for a review). In contrast, the findings in unconscious emotion processing are rather inconsistent. Some studies reported deficits in unconscious emotional processing in MDD [9]–[11] whereas others failed to find any changes in MDD [5]. For example, Mogg et al. [12] applied a Stroop task with emotional stimuli that were backward masked and thus unconsciously presented; MDD patients did not show any abnormalities in this task (see also [13]–[14]). Similarly, several studies could not observe an attention bias with masked emotional stimuli in depressed participants either [15]–[16]. A neuroimaging study [17] did not find any difference in right amygdala in response to backward-masked emotional faces between MDD patients and control subjects. In contrast, more recent analogous imaging studies did report deficits in the amygdala activity during masked faces [9]–[11]. Taken together, recent findings on unconscious emotional processing are inconsistent with regard to the unconscious deficits in MDD patients and it also remains unclear whether they are valence-specific.

Due to the fact that the commonly applied technique of backward masking yielded rather mixed results in unconscious emotional processing in MDD, we here adopted a different approach, continuous flashing interocular suppression (CFS) [18]–[20]. The CFS has been successfully and reliably used to tap into the implicit and automatic processing in the visual domain [21]–[22]. Compared to the backward-masking technique, the CFS can present information unconsciously throughout a relatively long viewing period (sometimes longer than 3 mins), potentially allowing for more robust and reliable unconscious processing [23]. In order to investigate the unconscious processing in the emotional domain, we combined the CFS with emotional faces of positive and negative valences. Using this paradigm, we conducted two experiments in healthy subjects using different stimulus durations (200 ms, 800 ms) of the viewing period (Experiments 1 and 2). This served as the basis for a third experiment (Experiment 3) where we directly compared healthy and MDD subjects in the emotional CFS to explore the valence-specific deficits in unconscious emotional processing in MDD.

## Methods

### Participants

All participants were recruited at Shanghai Mental Health Center. After a complete description of the study, written informed consent was obtained from each participant. The protocol of this study was approved by the Institute's Ethical Committees of both Shanghai Mental Health Center and Institute of Psychology, Chinese Academy of Sciences.

For Experiments 1 and 2, twenty healthy participants (13 females, 24.0±4.2 years old) were recruited according to following criteria: 13-term version of Beck Depression Inventory (BDI-13) ≤4; Self-rating Anxiety Scale (SAS)≤40; 17-term version of Hamilton Rating Scale for Depression (HAMD-17)≤7; 14-term version of Hamilton Rating Scale for Anxiety (HAMA-14)≤7; normal or corrected-to-normal vision (tested with international standard visual testing chart); no history of psychiatric or neurologic (or medial) disease; and no substance abuse. Both HAMD-17 and HAMA-14 have Chinese versions with good reliability and validity [24].

For Experiment 3, twenty-three inpatients with MDD diagnosed according to DSM-IV by two professional psychiatrists were recruited (13 females, 31.8±9.8 years old). Only subjects with BDI-13 score>7 and HAMD-17>7 were included. All the participants were cooperative during the test without severe suicidal ideation. The average HAMD score was 19.6 (SD = 9.3). MDD patients with severe anxious symptoms (Self-rating Anxiety Scale (SAS)>60, 14-item version of Hamilton Rating Scale for Anxiety (HAMA-14)>29) and/or other neurologic, psychiatric or medical disorders were excluded from the study. MDD patients were either medicated or not (serotoninergic drugs; see Table 1 for details).

**Table 1:** Clinical information summary of the participants.

		Medicated*MDD (n = 13)	UnmedicatedMDD (n = 8)	pooled MDD(n = 21)	Healthy Control(n = 22)
Age		33.0±11.2	29.9±6.6	31.8±9.7	30.2±10.1
Gender	Female	7	4	11	10
	Male	6	4	10	12
Mean Illness Duration (Yrs)		4.3±5.8	2.7±3.4	3.8±5.5	-
BDI		19.3±9.8	19.9±6.0	19.5±8.4	1.2±1.5
SAS		38.7±9.3	45.4±9.9	41.2±9.8	23.7±3.6
HAMD		19.5±11.1	19.8±5.9	19.6±9.3	0.3±1.1
HAMA		14.0±9.9	19.5±6.8	16.1±9.1	0.2±0.5

*Antidepressants (mg/d): Escitalopram(10); Sertraline(50); Paroxetine(20); Fluvoxamine(50); Fluoxetine(20); Venlafaxine(75); Citalopram(20); Mirtazapine(15); Doxepin (25); Trazodone (50); John's Wort Extracts (300).

In addition, twenty-three healthy control participants (HC, 13 females, 29.8±10.1 years old) matched for age and gender with the MDD patients were recruited according to the criteria in Experiment 1; this sample of healthy subjects was different from the one reported in Experiments 1 and 2. Demographic and clinical data for the MDD (further separated into medicated and un-medicated) and the HC groups were listed in Table 1 (Part of the information was obtained after data screening).

### Stimuli and Procedure

Stimuli were programmed with the psychophysical toolbox [25]–[26] on MATLAB (The MathWorks, Natick, MA), and were presented on a 17-inch flat-panel monitor. Two square frames (10.7°×10.7°) were displayed side by side on the screen. Through a mirror stereoscope mounted on a chinrest, each eye of the participant could only see the frame on the same side, and the mirror stereoscope was adjusted so that the two frames were comfortably fused together for the participant. The viewing distance was 40 cm (see [27] for setup of the equipment).

**Figure 1:**
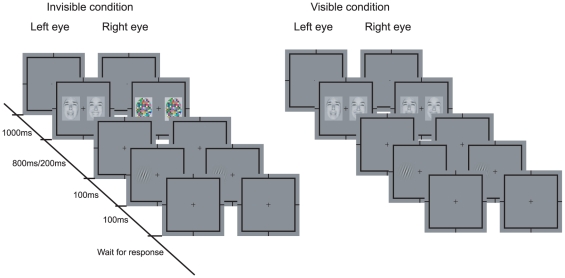
Schematic representation of the experimental paradigm for the invisible and visible conditions. In the invisible condition, dynamic noise patches were presented to the dominant eye and faces with happy and sad emotional expressions were presented to the other eye. The duration of the face presentation was 800 ms for Experiments 1 and 3, and 200 ms for Experiment 2. After a 100-ms interval, participants were instructed to press one of two buttons as soon and accurate as possible to indicate the perceived orientation (clockwise or counter-clockwise) of a Gabor patch presented for 100 ms. In the visible condition, the dynamic noise patches were replaced by the same pair of faces presented to the other eye.

The general experimental paradigm is shown in Figure 1. There were two separate sessions for invisible and visible stimuli, each containing 80 trials. At the beginning of each trial, a fixation cross (visual angle 0.8°×0.8°) was presented to each eye for one second. The following stimuli lasted for 800 ms in Experiments 1 and 3, and 200 ms in Experiment 2. The only difference between visible and invisible sessions was the content of the stimuli: In the invisible session, a pair of faces with different emotional expressions was presented to the participant's non-dominant eye (tested before experiment), while a pair of identical high contrast dynamic noise patches was presented to the dominant eye, so that the participant only perceived the identical noise patches due to the strong interocular suppression. For the visible session, a pair of faces with different emotional expressions was presented to both eyes and the participants could perceive the faces.

Each of the above face pairs was formed by grey-scale images of a positive and a negative emotional faces (4°×6° of visual angle), which were selected from happiness and sadness categories from three actors/actresses in Ekman and Friesen's [28] pictures of facial affect. The faces were masked using an ellipse so that the hair and background in the face images were excluded. The noise patches were masked in the same way to ensure their shape is the same as the face images. The distance between the centers of the two faces was around 5°. In half of the trials, the positive emotional faces were presented to the left, and the negative ones to the right side. For the other half trials, the positions were reversed.

The above stimuli were followed by a 100-ms fixation cross, and then identical small Gabor patches (2.5°×2.5°) were presented to both eyes for 100 ms. The positions of the Gabor patches were the centers of either the left or right previously presented faces. The Gabor patches were tilted one degree clockwise or counterclockwise (randomized), and the participant was instructed to press one of the two buttons (2-alternative force choice) to indicate their perception of the orientation of the Gabor patches, regardless of which side the Gabor patches were presented to. The fixation would not end until the participants made their choice. Among all 80 trials in each session, the presentation sides of the emotional faces and that of the Gabor patches were balanced so that there were 20 trials for each combination. The button-press and response time (RT) relative to the onset of the Gabor patch presentations was recorded.

Before the experiment, the participants were familiarized with the paradigm through a 50-trial training session. To ensure the participants were never explicitly aware of the invisible faces, they were instructed to press a different key to reject the trial if they detected grey-scale images during the invisible session. If more than two trials were rejected in the invisible session, the data for the corresponding participant were excluded from further analyses.

Experiments 1 and 2 were exactly the same except that the duration of the face presentation was 800 ms in Experiment 1 and 200 ms in Experiment 2. Experiment 3 was the same as experiment 1 but was applied to different participant populations, i.e., a new group of HC and a MDD group. For each session, the participants' responses were divided into two categories according to the emotional valence of the faces that were on the same side with the Gabor patches. The stimuli categories were defined as positive and negative emotion respectively. For each category, response accuracy (denoted as Acc_pos_ and Acc_neg_) was calculated by dividing the number of correct responses by the total number of valid trials. The trials with RT longer than 1500 ms or shorter than 100 ms were excluded. Within each session, the inter-category response accuracy difference, dAcc = Acc_pos_−Acc_neg_, was used as a measure of attention preference. A positive or negative value indicated attention preference to the positive or negative emotional faces respectively.

### Statistical analysis

In Experiments 1 and 2, the response accuracies for the positive and negative stimuli were separately tested against zero (using one-sample *t*-tests) for both the visible and the invisible conditions. The attention preference, as measured by dAcc = Acc_pos_−Acc_neg_, was further tested (using paired *t*-tests) between the invisible and visible conditions. In Experiment 3, before the above statistical analyses were conducted for each participant group, Chi-square tests were first performed to examine the gender and age difference between the two groups. After the within-group analyses (as used in Experiments 1 and 2), we further separated the MDD patients into medicated and unmedicated groups, and conducted a repeated-measure two-way ANOVA (visible/invisible×medicated/unmedicated) to examine the main effects and interactions between the two factors. Similarly, we conducted another repeated-measure two-way ANOVA (visible/invisible×HC/MDD) to examine the difference between the healthy control and MDD participants.

## Results

### Experiment 1 (800 ms): Healthy subjects

**Figure 2:**
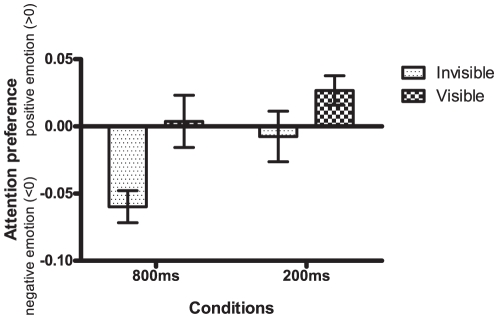
Comparison on attention preference in different sessions for healthy controls. The attention preference was indexed by difference in performance accuracy of the Gabor patch orientation judgment task. A positive value indicates attention preference to happy faces, and a negative value indicates attention preference to sad faces. In the 800-ms session, the invisible condition revealed significantly negative attention preference while the visible condition did not show significant valence preference. In the 200-ms session, no significant valence preference was observed in the invisible condition; the visible condition showed a positive trend though.

**Figure 3:**
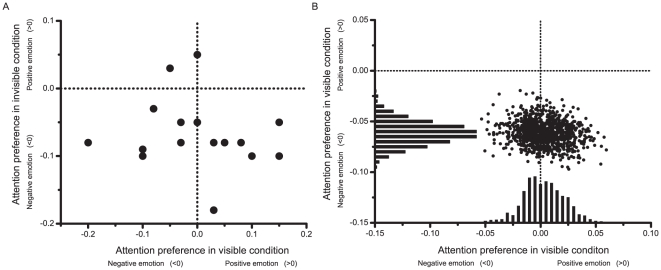
Individual attention preference of healthy participants in the invisible and visible conditions. (A) Scatter plot of individual attention preference across the invisible and the visible conditions. Each dot represents the attention preference for a participant. The locations of the dots are determined by the visible (horizontal axis) and the invisible (vertical axis) conditions. The zero levels that indicate no attention preference are illustrated using dotted-lines. Except two participants, all others show a negative attention preference in the invisible condition, but no such trend appears in the visible conditions. (B) Scatter plot of bootstrapped sample means of the attention preference. 1000 datasets are resampled from the original participants, each containing 20 participants, and the mean attention preference metrics for both invisible (horizontal axis) and visible conditions (vertical axis) for each resampled dataset are represented by a point on the scatter plot. The locations of the points are separately projected to the horizontal and the vertical axes, and the histograms are used to represent the distribution of the projected locations for the visible and the invisible conditions respectively. In the invisible condition, the distribution is below the zero level and has a relatively small deviation, indicating a robust negative bias in population level, while the distribution for the visible condition is centered at the zero.

No participants were excluded according to the criteria described in Methods. One-sample *t*-tests showed that the accuracy difference in the invisible session (dAcc_invisible_) was significantly lower than zero [*t*(19) = −4.964, *p* = .000], indicating observers' attentional preference for negative faces. This contrasted with the accuracy difference in the visible session (dAcc_visible_) that was not significantly different from zero [*t*(19) = .178, *p* = .867] thus showing no attentional bias for positive or negative valences. To further confirm the difference between visible and invisible sessions, we calculated the dAcc difference between both conditions and the result was significant [dAcc_invisible_−dAcc_visible_, *t*(19) = −2.595, *p* = .018] (see Figure 2). The difference is further demonstrated in the scatter plot of dAcc_invisible_ and dAcc_visible_ for individual participants (see Figure 3A). In addition, we used a standard bootstrapping procedure [29]–[30] to highlight the central tendency of dAcc_invisible_ and dAcc_visible_. Specifically, from the original participants, a bootstrapped sample with the same sample size (i.e., 20 participants) was nonparametrically resampled with replacement (i.e., a participant could be selected more than once). The averaged dAcc_invisible_ and averaged dAcc_visible_ from this bootstrapped sample was then plotted as a scatter plot (see Figure 3B). This procedure was repeated for 1000 times, and histograms representing population means and variations were generated for the dAcc_invisible_ and dAcc_visible_ respectively. The histogram of dAcc_invisible_ had a much higher kurtosis than that of dAcc_visible_ while the mode of the dAcc_invisible_ histogram was clearly lower than zero (See Figure 3B).

### Experiment 2 (200 ms): Healthy subjects

The same group of participants in Experiment 1 also participated in Experiment 2 which differed only in the presentation duration of the stimulus (200 ms instead of 800 ms). This experiment was undertaken to further examine whether the stimulus presentation duration modulates the unconscious effects observed in the invisible condition in Experiment 1. The dAcc_invisible_ was not significantly different from zero [*t*(19) = −.442, *p* = .663] while the dAcc_visible_ was significantly higher than zero [*t*(19) = 2.519, *p* = .021]. Hence, the shorter presentation time blurs the attentional preference to negative emotions in the unconscious mode while it yields a positive bias in the conscious mode (See Figure 2, 200 ms group). However, the difference between the invisible and visible sessions failed to achieve significance [dAcc_invisible_−dAcc_visible_, *t*(19) = −1.633, *p* = .119] (See Figure 2, 200 ms group).

### Experiment 3 (800 ms): Healthy and MDD subjects

Due to low response accuracy (lower than chance level), two MDD patients had to be excluded. In the HC group, one participant was excluded due to too few valid trials caused by abnormal response time. The age and gender ratio was matched between the two groups [age: *t*(41) = .524, *p* = .603, gender ratio: *χ* ^2^(1) = .020, *p* = .887]. The detailed demographic and clinical data are presented in Table 1.

A repeated-measure two-way ANOVA was performed to examine the difference between medicated and unmedicated MDD subjects in the dAcc in both experimental conditions, visible and invisible; this yielded no significant statistics in either main effects or interactions between medication groups and the experimental conditions [*F*(1, 19) = .17, *p* = .684 for main effect of the experimental conditions, *F*(1, 19) = .325, *p* = .575 for experimental condition×medication group interaction]. This indicated the medication did not play an important role in the results, and therefore the two MDD groups were merged for further analyses.

The dAcc difference between the MDD and HC groups across two experimental conditions (visible and invisible) was examined by a repeated-measure two-way ANOVA. The results showed a significant interaction effect between the experimental conditions and the subject groups [*F*(1, 41) = 4.537, *p* = .039].

**Figure 4:**
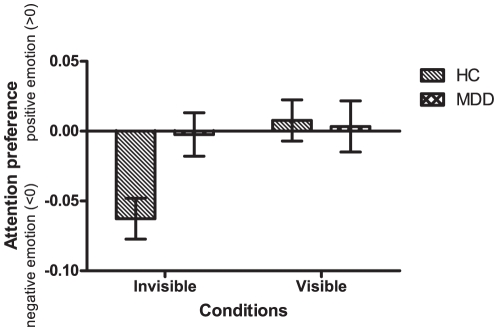
Comparisons of attention preference between healthy controls and MDD patients. In the invisible condition, the healthy controls showed significantly negative attention preference, but the MDD patients did not. The difference between the two groups was significant. In the visible condition, the two groups behaved similarly.

Further simple effect analysis showed significant dAcc difference between MDD and HC groups in the invisible condition [*F*(1, 41) = 7.900, *p* = .008, see Figure 4]. The dAcc_invisible_ for the MDD group was not significantly different from zero [*t*(20) = −.153, *p* = .880], indicating that there was no bias between positive and negative valences in MDD patients in the invisible condition. In contrast, the dAcc_invisible_ for the HC group was significantly negative [*t*(21) = −4.296, *p* = .000], which was consistent with the findings in Experiment 1.

In the visible condition, however, there was no significant dAcc_visible_ difference between HC and MDD groups [*F*(1, 14) = .030, *p* = .874, see Figure 4]. Specifically, the dAcc_visible_ for the MDD groups was not significantly different from zero [*t*(20) = −4.296, *p* = .864], and so was the dAcc_visible_ for the HC groups [*t*(21) = .507, *p* = .618].

**Figure 5:**
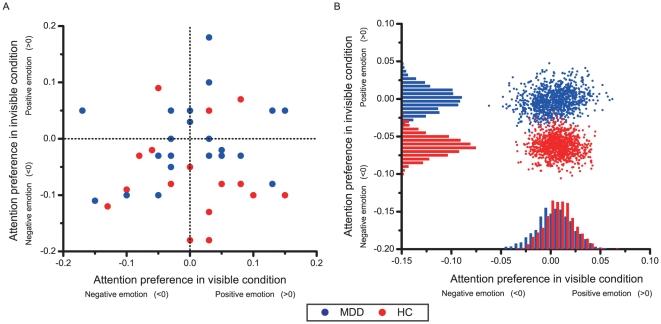
Individual attention preference of healthy controls and MDD patients. (A) Scatter plot of attention preference of MDD patients (blue points) and healthy controls (red points) across the visible (horizontal axis) and the invisible (vertical axis) conditions. The horizontal and vertical dash lines represent the no-preference level for the visible and invisible conditions respectively. Most healthy controls showed negative attention preference in the invisible condition (most red points are below horizontal dash line), but there was no such trend in MDD patients (the blue points show no obvious trend relative to the horizontal dash line). (B) Scatter plot of bootstrapped sample means of the two groups. For each participant group, 1000 datasets (with the same number of participants of the original group) are resampled from the original participants, and the mean attention preference metrics for both invisible and visible conditions for each dataset are represented by locations of the points on the scatter plot. The locations are projected to horizontal and vertical axes, based on which the distributions of attention preference metrics for the visible and invisible conditions are generated. The distribution for the MDD patients (blue histograms) and the healthy controls (red histograms) are clearly separated in the invisible condition but mixed together in the visible condition, showing a good separation between the two participant groups in the invisible paradigm.

Similarly, we presented scatter plots for individual participants as in Experiment 1 to demonstrate that the difference between the MDD and HC groups in the invisible condition was more robust than in the visible condition (see Figure 5A). To highlight the inter-group difference on dAcc_invisible_, bootstrap resampling procedures similar to that used in Experiment 1 were conducted within each of the groups (see Figure 5B). The histograms representing population means and variations showed significant difference between the two groups in the invisible condition but not in the visible condition.

## Discussion

This study investigated unconscious positive and negative emotion processing in both clinical MDD patients and healthy controls. The main findings are: 1) Healthy subjects preferred to attend to positive emotional-valence stimuli in the visible, i.e., conscious condition, while negative valences significantly attracted more attention in the invisible, i.e., unconscious condition; and 2) the unconscious attention preference to negative emotional valences remained absent in MDD patients which allowed to clearly distinguish them from the healthy group.

### Valence-specific unconscious emotional processing in healthy subjects

Healthy subjects showed attention preference to sad facial expression in the unconscious condition (Experiment 1). This is in accordance with various studies showing that emotional contents as distinguished from non-emotional ones are preferentially processed in unconscious condition. For example, Dimberg et al. [31] found that both positive and negative emotional reactions can be unconsciously evoked (also [32]); Jiang and He [33] showed that amygdala activity could be induced by fearful but not neutral facial expressions during unconscious presentation. What remains unclear though is whether this preference for emotions in the unconscious mode is valence-specific, meaning whether it pertains specifically for negative or positive emotions.

**Figure 6:**
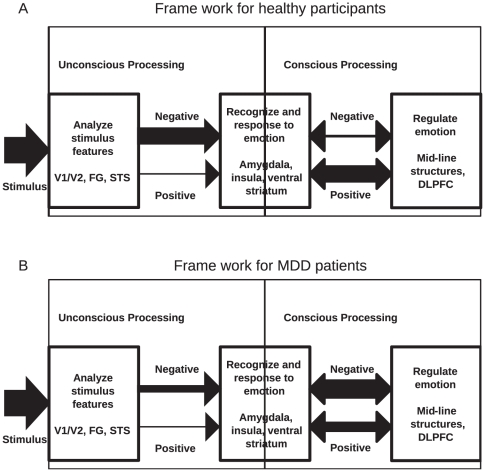
Conceptual schema combining the implications of the current findings into a general framework. As proposed by numerical studies, the emotional stimuli are processed through three general phases, including analysis of stimulus features, recognition and response to emotion, and emotion regulation. The processing can be separated into conscious and unconscious parts. (A) Framework for healthy participants. Studies on conscious emotion processing have suggested a positive preference in healthy subjects [39], we therefore mark the positive processing in conscious processing using a bold arrow and the negative processing using a narrow arrow. Our findings indicate that in the unconscious processing stage, the negative emotions may be processed through a stronger and faster pathway, as indicated by the bold arrow. (B) Framework for MDD patients. Our findings also suggest that MDD patients may have a deficit in unconscious negative emotion processing (see the thinner arrow for negative emotion in the unconscious part). This, in turn, may affect the conscious processing which then becomes shifted from positive to negative emotion preference (see the bolder arrow for negative emotion in the conscious part) in order to compensate the hitherto incomplete processing of negative emotions in the deficient unconscious mode.

Our findings in healthy subjects demonstrated that processing in especially the unconscious mode is valence-specific by showing the specific impact of negative emotions. However, such preference for negative emotions was not observed in the conscious mode where the opposite valence, positive emotions, dominated. This raises the question for possible mechanisms of this preference for negative emotions in the unconscious mode. One may assume that negative emotions are processed with stronger and possibly also faster than positive emotions in the unconscious mode. This may also be neurobiologically plausible given that unconscious processing of negative emotions has been specifically associated with direct fast subcortical pathways from the visual cortex to the amygdala (see [34], [35] for a review). In contrast, conscious processing has been related to relatively slower connections from the amygdala to the prefrontal cortex (for a review, see [5]) (see Figure 6a). However, this assumption remains speculative at this point awaiting further supporting evidence from future functional imaging studies.

In contrast to the preference for negative emotions in the unconscious mode, we observed a preference for positive emotions in the conscious mode in Experiment 2. This is well in accordance with previous studies that also observed a similar positive attention bias in the conscious condition in healthy participants [36]–[39].

It should be noted though that we obtained such positive preference only in our Experiment 2 whereas it was not observed in Experiment 1. We suspected that this was due to the 800-ms cue duration as adopted and optimized for the unconscious condition [18], [27]. Due to this rather long duration, participants may have directed their attention away from the stimuli in the conscious condition thereby preventing the positive emotions form exerting their full effect. In order to avoid this problem, we reduced the exposure duration to 200 ms in Experiment 2. As a result, the positive bias in the visible condition was observed as expected, which though impact the unconscious condition and its preference for negative emotions in a negative way. Though seemingly a merely methodological problem, the impact of the duration time on the interaction with positive and negative emotions in both unconscious and conscious modes may point to an underlying neurophysiological mechanism. More specifically, our results suggest that the temporal duration of the stimulus may be central for inducing the neurophysiological mechanisms underlying the valence-specific effects in both unconscious and conscious modes. This remains to be investigated in the future.

### Disturbed negative emotional unconscious processing in depressed patients

In contrast to the healthy subjects, MDD patients did not show any preference towards negative emotions in the invisible condition, i.e., the unconscious mode. Hence, their automatic and implicit negative emotional processing seems to be diminished in the unconscious mode. This complements and extends the many findings showing a negative bias in conscious emotion processing in for instance emotion regulation and attention tasks [40]–[42]. Taken together, these findings support the proposed hypothesis that depressed individuals may not automatically (and thus unconsciously) orient their attention towards negative information in the environment, but once such information has come to be the focus of their attention, they may have greater difficulty disengaging from it [5].

Our results may also be clinically relevant in that unconscious negative emotional processing may provide a novel and more viable target for future psychotherapeutic and/or pharmacotherapeutic intervention than conscious emotion regulation strategies. More specifically, it means that we have to target unconscious processing rather than conscious processing as targeted in Cognitive Behavioral Therapy. Hence, our hypothesis, if confirmed in the future, may stipulate the development of more specific psychotherapeutic strategies.

The same obviously holds for pharmacotherapeutic strategies. As discussed above, unconscious processing is mediated predominantly by fast processing in subcortical systems, while conscious processing is rather related to slower processing in cortical regions. Interestingly, depressed patients indeed show major resting state abnormalities in these subcortical regions ranging from the brain stem (raphe nucleus, locus coerulus) to the dorsomedial thalamus, the PAG, the tectum, the colliculi, and the amygdala (and ultimately to the cortex [6], [9]–[11], [17], [43]–[45]).

How are the resting state abnormalities in these regions related to the observed deficits in unconscious negative emotional processing? The abnormally high resting state activity [46] may affect first and predominantly the primary and fast unconscious processing of emotional stimuli while it may exert less impact on the slower conscious processing. One would hence assume abnormally decreased rest-stimulus interaction [47] in subcortical midline regions in MDD during especially negative emotional processing. Such decreased rest-stimulus interaction may then be manifested in decreased preference for negative emotions in the unconscious mode and that is exactly what we observed in our current study. Based on our findings, one may envision the developments of pharamcotherapeutic strategies (and other therapeutic tools like deep brain stimulation) that specifically target the subcortical fast tracks and their rest-stimulus interaction rather than the cortical slow connections. This however remains a scenario for the future.

How the deficits in unconscious negative processing are related to the conscious processing of emotions? Depressed patients show a deficit in unconscious emotion processing which may let them puzzled about the relevance and significance of negative emotions; this in turn may induce the often described emotion regulation deficits and the abnormal attention bias towards negative emotions in the conscious mode (see Figure 6b).

In conclusion, we here demonstrate for the first time negative valence-specific effects in unconscious emotion processing in healthy subjects and their disruption in MDD patients. This yields not only novel insights into unconscious processing in general but suggests also that the often observed negative bias in the conscious mode in MDD may result from a deficit in unconscious negative processing.
